# Supplementary material for: Thirteen dubious ways to detect conserved structural RNAs
Source: IUBMB Life. Author manuscript; Available in PMC 2024 Jul 10. (PMC11234323; doi:10.1002/iub.2694)

cmf.M0662940_ext_liftover.hg38.vertebrates_90.Eval_1e-10_1.cacofold

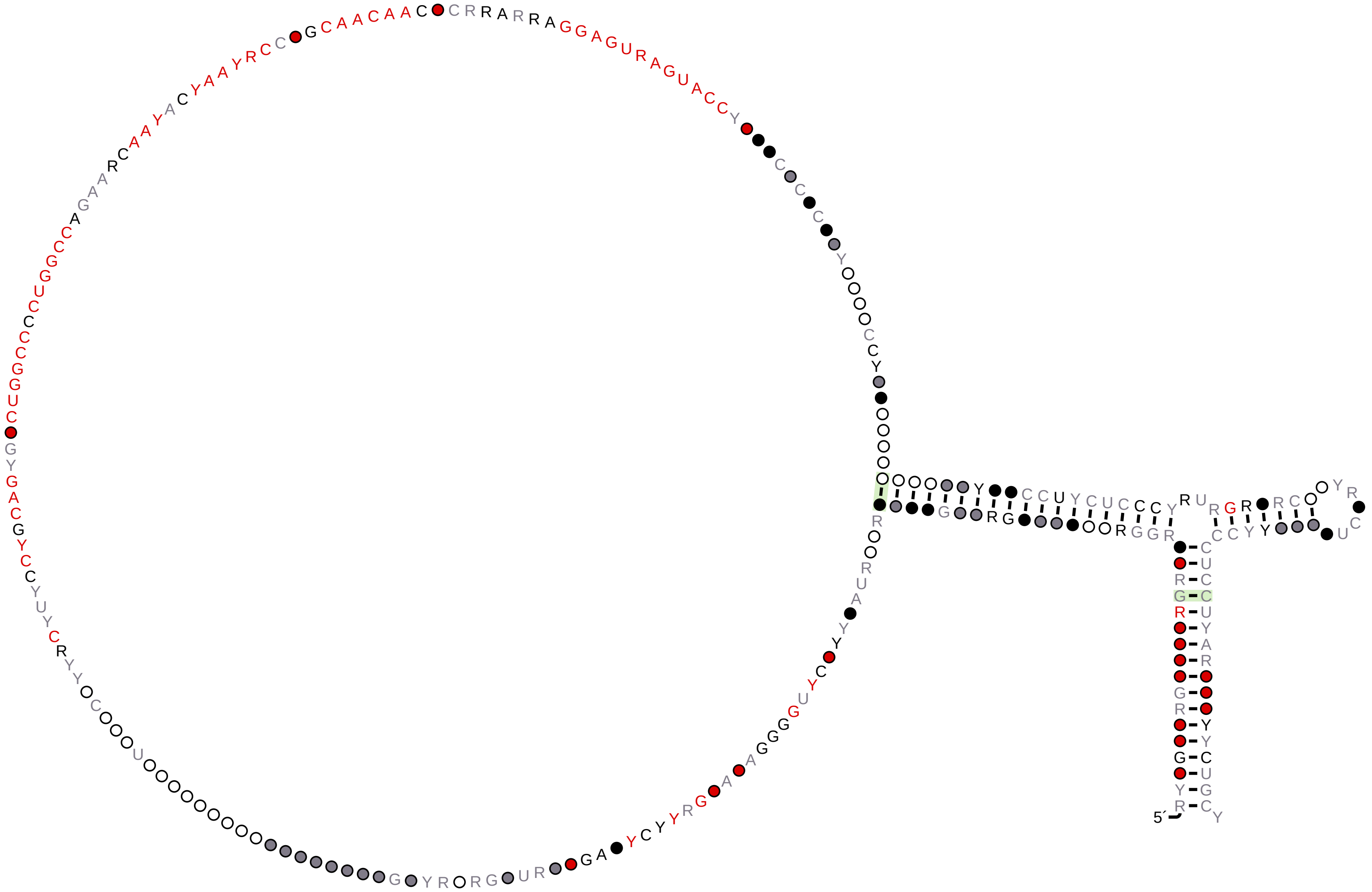

Supplement: supplementary_material [file NIHMS1907110-supplement-supplementary_material.gz › supplemental_material/Table1/CRS.structural.tar.gz!/selected/selected_noRfam/noNUMTs/cmf.M0662940/MyM0662940/R-scape_cmsearch/cmf.M0662940_ext_liftover.hg38.vertebrates_90.Eval_1e-10_1.cacofold.R2R.sto.pdf]
